# Heterogeneous response to TGF-β1/3 isoforms in fibroblasts of different origins: implications for wound healing and tumorigenesis

**DOI:** 10.1007/s00418-023-02221-5

**Published:** 2023-09-14

**Authors:** Lukáš Urban, Matúš Čoma, Lukáš Lacina, Pavol Szabo, Jana Sabová, Tomáš Urban, Hubert Šuca, Štefan Lukačín, Robert Zajíček, Karel Smetana, Peter Gál

**Affiliations:** 1 Department of Pharmacology, Faculty of Medicine, Pavol Jozef Šafárik University in Košice, 040 11 Košice, Slovak Republic; 2 Department of Biomedical Research, East-Slovak Institute of Cardiovascular Diseases Inc, Ondavská, 040 11 Košice, Slovak Republic; 3 Institute of Anatomy, First Faculty of Medicine, Charles University, U Nemocnice 2, 128 00 Prague, Czech Republic; 4 BIOCEV, First Faculty of Medicine, Charles University, 252 50 Vestec, Czech Republic; 5 Department Dermatovenereology, First Faculty of Medicine, Charles University and General University Hospital, 128 08 Prague, Czech Republic; 6 Prague Burn Center, Third Faculty of Medicine, Charles University and University Hospital Královské Vinohrady, 100 00 Prague, Czech Republic; 7 Department of Heart Surgery, East-Slovak Institute of Cardiovascular Diseases Inc, 040 11 Košice, Slovak Republic; 8 Department of Pharmacognosy and Botany, Faculty of Pharmacy, Comenius University, 832 32 Bratislava, Slovak Republic; 9 Institute of Neurobiology, Biomedical Research Center of the Slovak Academy of Sciences, 040 01 Košice, Slovak Republic

**Keywords:** Tumor microenvironment, Keloid, Hypertrophic scar, Melanoma, Carcinoma, Stroma

## Abstract

Identification of therapeutic targets for treating fibrotic diseases and cancer remains challenging. Our study aimed to investigate the effects of TGF-β1 and TGF-β3 on myofibroblast differentiation and extracellular matrix deposition in different types of fibroblasts, including normal/dermal, cancer-associated, and scar-derived fibroblasts. When comparing the phenotype and signaling pathways activation we observed extreme heterogeneity of studied markers across different fibroblast populations, even within those isolated from the same tissue. Specifically, the presence of myofibroblast and deposition of extracellular matrix were dependent on the origin of the fibroblasts and the type of treatment they received (TGF-β1 vs. TGF-β3). In parallel, we detected activation of canonical signaling (pSMAD2/3) across all studied fibroblasts, albeit to various extents. Treatment with TGF-β1 and TGF-β3 resulted in the activation of canonical and several non-canonical pathways, including AKT, ERK, and ROCK. Among studied cells, cancer-associated fibroblasts displayed the most heterogenic response to TGF-β1/3 treatments. In general, TGF-β1 demonstrated a more potent activation of signaling pathways compared to TGF-β3, whereas TGF-β3 exhibited rather an inhibitory effect in keloid- and hypertrophic scar-derived fibroblasts suggesting its clinical potential for scar treatment. In summary, our study has implications for comprehending the role of TGF-β signaling in fibroblast biology, fibrotic diseases, and cancer. Future research should focus on unraveling the mechanisms beyond differential fibroblast responses to TGF-β isomers considering inherent fibroblast heterogeneity.

## Introduction

Fibroblasts are transcriptionally and functionally heterogeneous cell populations of mesenchymal origin participating in the production and homeostasis of connective tissue (Lynch and Watt 2018). They produce various structural macromolecules and proteolytic enzymes responsible for the deposition and remodeling of extracellular matrix (ECM), thus when activated aberrantly supporting fibrosis (Talbott et al. 2022). When differentiated into myofibroblasts, a highly contractile α-smooth muscle actin+ (α-SMA+) phenotype (Hinz et al. 2007), fibroblasts represent an important cell population contributing to (but are not necessary for) wound contraction (Ibrahim et al. 2015). Cancer-associated fibroblasts (CAFs) resemble the myofibroblast-like phenotype modulating the biological properties of tumors (Nurmik et al. 2020). Apart from ECM production, fibroblasts also form a signaling niche. Depending on the origin and acquired phenotype, (myo)fibroblasts produce several cytokines/chemokines, growth factors, and additional signaling molecules (Coma et al. 2021; Gal et al. 2017) regulating clinically relevant parameters of tumor/wound microenvironment (TME/WME). Interestingly, isolated fibroblasts maintain regional information and distinct cellular progeny (Jarkovska et al. 2014; Dvorankova et al. 2012; Szabo et al. 2011; Mateu et al. 2016).

The model of critical depth of injury represents an illustrative example of two functionally and morphologically distinct skin fibroblast populations with a crucial impact on wound healing outcomes. While superficial injury targeting the papillary dermis results in normotrophic almost invisible scar formation, deeper wounds reaching the reticular dermis often activate prolonged inflammatory responses resulting in hypertrophic or keloid scarring (Coma et al. 2021). Detailed single-cell sequencing (SCS) of keloid scars revealed the presence of four fibroblast subpopulations: (i) secretory-papillary, (ii) secretory-reticular, (iii) mesenchymal, and (iv) pro-inflammatory (Deng et al. 2021). In parallel, SCS of tumor stroma revealed at least three functionally unique populations of CAFs acting within the TME as shown in breast or pancreatic cancers (Kieffer et al. 2020; Elyada et al. 2019): (a) ECM-producing high-α-SMA/low-cytokine (TGF-β-responsive) myofibroblastic CAFs (myCAFs), (b) pro-inflammatory low-α-SMA/high-cytokine (interleukin (IL)-6/IL-11) CAFs (iCAFs), and (c) antigen-presenting CAFs (apCAFs). The genomic approach further pointed out the potential of CAF signatures to act as a prognostic indicator, e.g., in head and neck squamous cell carcinoma (Yang et al. 2022).

Out of several wound healing regulatory molecules, TGF-β may be considered a critical element with the broadest spectrum of effects driving ECM deposition, fibrosis, and epithelial-to-mesenchymal transition (EMT) (Bielefeld et al. 2013; Hao et al. 2019). Recent discoveries showed that, while formerly conceived as a simple non-amplified signaling pathway, TGF-β signaling is better represented by complex signaling including canonical (SMAD2/3) and/or non-canonical (Erk1/2, JNK, p38, Akt, ROCK) (Zhang 2017) pathways with remarkable importance also in fibrosis (Trojanowska 2009). The isoforms TGF-β1, TGF-β2, and TGF-β3 play important roles in wound contraction and scarring. Among them, TGF-β1 is the most prevalent and biologically relevant, and has been linked to excessive scarring and fibrosis (Klass et al. 2009; Shi et al. 2014). TGF-β3 shares sequence homology with TGF-β1, but has been shown to have anti-fibrotic effects on skin tissue and is more prevalent in fetal (scarless) wound healing (Hsu et al. 2001).

We conducted a study to explore the cellular response of fibroblasts isolated from different neoplastic, healing, and normal tissues to TGF-β, which is a hallmark signaling molecule of fibrosis. Our investigation focused on the activation of TGF-β1 and TGF-β3 signaling pathways, as well as the resulting changes in the induced phenotype shifts. This research was motivated by the similarities between wound healing and tumor progression (Dvorak 1986, 2015), and we aimed to gain insights into the molecular mechanisms that underlie these processes.

## Materials and methods

### Human primary cultures of cells and tissue sample collection

Human dermal fibroblasts (HDFs, *n* = 2) were isolated from healthy donors undergoing reduction mammaplasty at the Department of Plastic Surgery, Third Faculty of Medicine and Kralovske Vinohrady University Hospital in Prague. Pancreatic ductal adenocarcinoma (PDAC) fibroblasts (PANFs, *n* = 2) were isolated from pancreatic cancer tissue samples obtained from the Department of Pathology, Third Faculty of Medicine, Charles University and University Hospital Kralovské Vinohrady in Prague. Squamous cell carcinoma fibroblasts (SCCFs, *n* = 2) were isolated from squamous cell carcinoma located in the root of the tongue at the Department of Stomatology, General University Hospital in Prague. Metastasis of breast cancer fibroblasts (BCMF, *n* = 1) were isolated from skin metastasis of breast cancer, and basal cell carcinoma fibroblasts (BCCFs, *n* = 1) were isolated from basal cell carcinoma covering the skin of an upper limb at the Department of Dermatology and Venerology, First Faculty of Medicine, Charles University. Fibroblasts from keloid (KSF, *n* = 1), active hypertrophic (ASF, *n* = 1) and quiescent scars (QSF, *n* = 1) were isolated at Prague Burn Centre, Third Faculty of Medicine, Charles University and University Hospital Královské Vinohrady, Czech Republic.

Fibroblasts were isolated and cultured following a previously described procedure (Dvorankova et al. 2019) with informed consents of the patients respecting the Declaration of Helsinki and approved by local ethical committees. Briefly, small pieces of skin/cancer/wound tissue samples were trypsinized (0.25%, 15 min, RT) (Sigma-Aldrich, St. Louis, MO, USA) and seeded into 6-well plates covered with Dulbecco’s Modified Eagle Medium (DMEM) containing 10% FBS and 2% antibiotics/antimycotics (penicillin/streptomycin/gentamicin/amphotericin B) (all from Biochrom, Berlin, Germany) at 37 °C and 5% CO_2_/95% air atmosphere. After few days, the migrating fibroblasts were collected into cultivation flask and expanded by further culturing. Vimentin-expressing cells negative for CD45 (leukocyte marker), keratins (epithelial marker), and CD31 (endothelial marker) were considered fibroblasts.

### Western blot (WB) analysis

Primary cultures of fibroblasts (passages 9–11) were seeded on Petri dishes at the density of 5000 cells/cm^2^ and cultivated for 7 days in the presence (10 µg/mL) or absence (control) of TGF-β1 and TGF-β3 (both PeproTech, London, UK).

Protein lysates were prepared as follows: cells were scratched and lysed in Laemmli lysis buffer [0.1 M Tris/HCl (pH 6.8), 20% glycerol, 10% SDS (sodium dodecyl sulfate)] completed with protease and phosphatase inhibitors (Sigma-Aldrich, St. Louis, MO, USA) and disrupted by sonication (QSonica, 40% amplitude, 15 s).

Protein concentration of samples was carried out using a Pierce® BCA Protein Assay Kit (Thermo Scientific, Rockford, IL, USA) and measured at Cytation™ 3 Cell Imaging Multi-Mode Reader (Biotek, Winooski, VT, USA). Following a quick boiling step (95 °C, 5 min), 10 µg of each sample was loaded into 10% Bis-Tris SDS-PAGE. After separation, proteins were electroblotted onto PVDF membrane using iBlot 2 (Thermo Fisher Scientific, Waltham, MA, USA). Afterwards, membranes were blocked (1 h, RT) in 5% NFDM/BSA (non-fat dry milk/bovine serum albumin) dissolved in TBST (tris-buffered saline with 0.1% Tween) and incubated with primary antibody overnight at 4 °C on tube rollers. The next day, membranes were washed three times with TBST and incubated with HRP-conjugated secondary antibody (1 h, RT). Protein presence was detected as chemiluminescent signal using ECL (SuperSignal West Pico PLUS Chemiluminescent Substrate, Thermo Fisher Scientific, Waltham, MA, USA) acquired at iBright FL1500 Imaging System (Thermo Fisher Scientific, Waltham, MA, USA). Even sample loading was assessed by β-actin staining. The set of primary and secondary antibodies applied in the analysis are shown in Table 1.

**Table 1:** Reagents used for western blot analysis

	Host	Isotype	Clonality	Manufacturer
Primary antibody				
Vimentin	Rabbit	IgG	Monoclonal	CST, Danvers, MA, USA
HMW	Mouse	IgG	Monoclonal	Thermo Fisher Scientific Inc., Waltham, MA, USA
α-SMA	Rabbit	IgG	Monoclonal	CST, Danvers, MA, USA
F-actin	Mouse	IgM	Monoclonal	Abcam, Cambridge, UK
Fibronectin	Rabbit	IgG	Monoclonal	Abcam, Cambridge, UK
Vinculin	Rabbit	IgG	Monoclonal	Abcam, Cambridge, UK
Tenascin C	Rabbit	IgG	Monoclonal	CST, Danvers, MA, USA
PDGFR-α	Rabbit	IgG	Monoclonal	CST, Danvers, MA, USA
PDGFR-β	Rabbit	IgG	Monoclonal	CST, Danvers, MA, USA
FAP	Rabbit	IgG	Polyclonal	Abcam, Cambridge, UK
PDPN	Mouse	IgG	Monoclonal	Abcam, Cambridge, UK
TGM2	Mouse	IgG	Monoclonal	Abcam, Cambridge, UK
IL-6	Rabbit	IgG	Monoclonal	CST, Danvers, MA, USA
pSMAD 2/3	Rabbit	IgG	Monoclonal	CST, Danvers, MA, USA
SMAD 2/3	Rabbit	IgG	Monoclonal	CST, Danvers, MA, USA
β-catenin	Rabbit	IgG	Monoclonal	CST, Danvers, MA, USA
pAKT	Rabbit	IgG	Monoclonal	CST, Danvers, MA, USA
AKT	Rabbit	IgG	Polyclonal	CST, Danvers, MA, USA
pERK 1/2	Rabbit	IgG	Polyclonal	CST, Danvers, MA, USA
ERK 1/2	Rabbit	IgG	Monoclonal	CST, Danvers, MA, USA
pRAF	Rabbit	IgG	Monoclonal	Abcam, Cambridge, UK
RAF	Rabbit	IgG	Polyclonal	Abcam, Cambridge, UK
HRAS	Rabbit	IgG	Polyclonal	Abcam, Cambridge, UK
KRAS	Rabbit	IgG	Polyclonal	Abcam, Cambridge, UK
ROCK1	Rabbit	IgG	Monoclonal	CST, Danvers, MA, USA
MLCK	Rabbit	IgG	Polyclonal	Thermo Fisher Scientific Inc., Waltham, MA, USA
β-actin	Rabbit	IgG	Monoclonal	CST, Danvers, MA, USA
Secondary antibody				
Anti-rabbit IgG, HRP-linked	Goat	IgG		CST, Danvers, MA, USA
Anti-mouse IgG, HRP-linked	Horse	IgG		CST, Danvers, MA, USA

### Immunofluorescence (IF) analysis

Fibroblasts at passages 9–11 were seeded on coverslips at a density of 2000 cells/cm^2^ and cultured for 7 days in the presence (10 ng/mL) or absence (control) of the tested TGF-β1 and TGF-β3 (both PeproTech, London, UK).

Cells were fixed with 2% buffered paraformaldehyde (pH 7.2) for 5 min and washed with phosphate-buffered saline (PBS). Cell membranes were permeabilized using Triton X-100 (Sigma-Aldrich, St. Louis, MO, USA), and sites for the antigen-independent binding of antibodies were blocked with porcine serum albumin (DAKO, Glostrup, Denmark). The commercial antibodies were diluted according to the manufacturer’s instructions and the specific set of antibodies used for immunofluorescent analysis are listed in Table 2. To ensure the specificity of the immunocytochemical staining, replacement of the first-step antibody with an irrelevant antibody was done and positive control samples were tested. Nuclear staining was performed using 4′,6-diamidino-2-phenylindole (DAPI; Sigma-Aldrich, St. Louis, MO, USA). All samples were mounted in Vectashield (Vector Laboratories, Burlingame, CA, USA) and inspected using an Eclipse 90i fluorescence microscope (Nikon, Tokyo, Japan) equipped with filter cubes for FITC, TRITC, and DAPI, and a digital camera C11440 ORCA-flash 4.0 (Hamamatsu, Hamamatsu City, Japan) with NIS-Elements computer-assisted image analysis software (Nikon, Tokyo, Japan).

**Table 2:** Reagents used for immunofluorescence

	Host	Isotype	Clonality	Manufacturer
Primary antibody				
Smooth muscle actin	Mouse	IgG	Monoclonal	Dako, Glostrup, Denmark
Vimentin	Mouse	IgG	Monoclonal	Dako, Glostrup, Denmark
Fibronectin	Rabbit	IgG	Polyclonal	Dako, Glostrup, Denmark
Tenascin C	Rabbit	IgG	Monoclonal	CST, Danvers, MA, USA
Secondary antibody				
Anti-rabbit IgG, Alexa Fluor™ Plus 488	Goat	IgG	Polyclonal	Invitrogen, Carslbad, CA, USA
Anti-mouse IgG, Alexa Fluor™ Plus 488	Goat	IgG	Polyclonal	Invitrogen, Carslbad, CA, USA
Anti-rabbit IgG, Alexa Fluor™ Plus 594	Goat	IgG	Polyclonal	Invitrogen, Carslbad, CA, USA
Anti-mouse IgG, Alexa Fluor™ Plus 594	Goat	IgG	Polyclonal	Invitrogen, Carslbad, CA, USA

## Results

### Western blot analysis of fibroblast phenotype

**Fig. 1:**
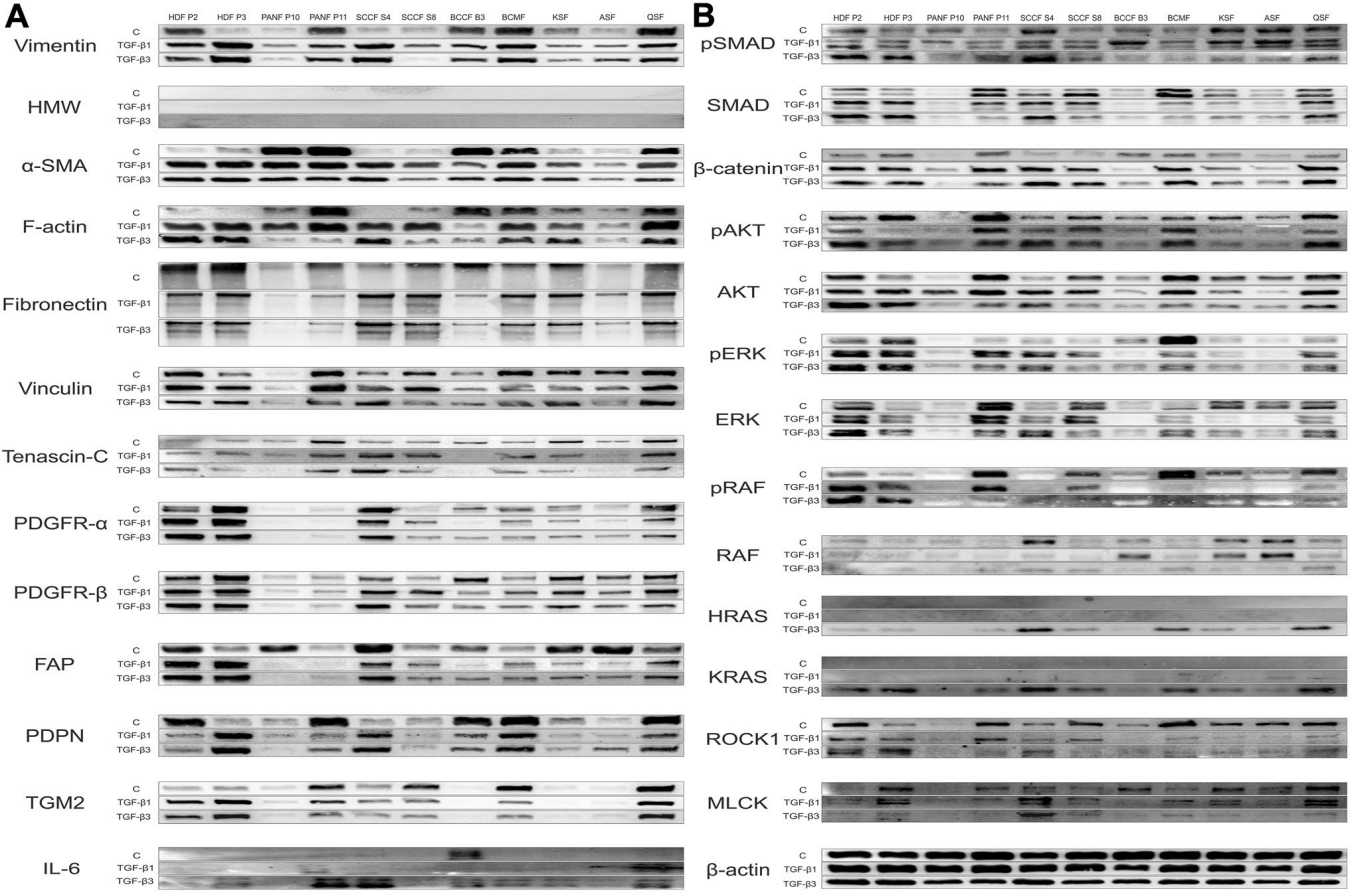
Western blot analysis of studied fibroblasts. Protein expression analysis in cultures of human dermal fibroblasts (HDFs), pancreatic ductal adenocarcinoma fibroblasts (PANFs), squamous cell carcinoma fibroblasts (SCCFs), basal cell carcinoma fibroblasts (BCCFs), breast cancer metastasis fibroblasts (BCMFs), keloid scar fibroblasts (KSFs), active/hypertrophic scar fibroblasts (ASFs), and quiescent scar fibroblasts (QSFs). **a** Protein expression profile of cell phenotype: vimentin, high molecular weight cytokeratin (HMW), α-smooth muscle actin (α-SMA), F-actin, fibronectin, vinculin, tenascin C, platelet-derived growth factor alpha (PDGFR-α), platelet-derived growth factor beta (PDGFR-β), fibroblast activation protein (FAP), podoplanin (PDPN), transglutaminase 2 (TGM2), interleukin-6 (IL-6). **b** Protein expression profile of cell signaling: phospho/small mothers against decapentaplegic 2/3 (pSMAD2/3, SMAD2/3), β-catenin, phospho/Akt (p/AKT, AKT), phospho/extracellular signal-regulated kinase 1/2 (pERK, ERK). phospho/rapidly accelerated fibrosarcoma (pRAF, RAF), Harvey Rat sarcoma virus GTPase (HRAS), Kirsten rat sarcoma virus GTPase (KRAS), rho-associated, coiled-coil-containing protein kinase 1 (ROCK1), myosin light-chain kinase (MLCK). β-actin was used as sample loading control

We analyzed the expression profile of fibroblasts cultivated in control medium and in medium supplemented with either TGF-β1 or TGF-β3. Representative photographs from the WB analysis are shown in Fig. 1a.

Our analysis revealed the presence of vimentin expression in all types of fibroblasts with varying levels of intensity. TGF-β1 and TGF-β3 were able to stimulate vimentin expression in some studied fibroblasts with no effect on KSF/ASF that remained weakly positive. None of the cells were positive for HMW, indicating their mesenchymal phenotype (not origin).

Notably, we observed high expression of α-SMA, indicative of myofibroblasts, in primary cultures of PANFs, BCCFs, BCMFs, and QSFs under control conditions (TGF-β-free medium). Subsequent treatment with TGF-β1 or TGF-β3 led to strong upregulation of α-SMA expression, primarily in primary cultures that initially exhibited low α-SMA expression.

Intriguingly, CAFs and KSFs/ASFs exhibited lower levels of fibronectin, structural glyocoprotein of the ECM, compared to HDFs and QSFs. Furthermore, treatment with TGF-β1 or TGF-β3 resulted in increased fibronectin expression in SCCFs only, while fibronectin expression in BCCFs was rather diminished (not observed in IF). We observed tenascin C, another important component of the ECM, expression in CAFs and scar fibroblasts with only negligible changes following TGF-β1 and TGF-β3 treatment.

PDGFR-β was highly expressed in skin-derived (HDFs and scar) fibroblasts, whereas high expression of PDGFR-α was not observed in KSFs/ASFs. Of note the expression of PDGFR varied between studied CAFs. TGF-β1 or TGF-β3 treatments had only weak/no effects on their expression across studied cells.

Interestingly, TGF-β1 and TGF-β3 were able to decrease FAP expression in KSFs/ASFs and in PANF P10 which were strongly positive only for α-SMA out of other studied proteins.

Our study uncovered an interesting finding that TGF-β1 and TGF-β3 were able to decrease expression of FAP in both KSFs/ASFs and in PANF P10. In contrast to other studied proteins, the PANF P10 cells were strongly positive only for α-SMA.

We observed highly variable expression levels of several proteins, including PDPN, TGM2, and F-actin, among the studied fibroblasts. While no differences in TGF-β1 or TGF-β3 stimulation were detected in skin-derived fibroblasts, the expression levels of these proteins varied in CAFs. In particular, the F-actin levels observed in CAFs underscore remarkable differences between TGF-β1 vs. TGF-β3 response. Intriguingly, TGF-β1/3 treatment was able to switch the PDPN phenotype from positive to negative, or vice versa. Of note, the levels of vinculin expression remained largely stable within the studied populations of fibroblasts following treatment with TGF-β1/3.

Importantly, BCCF B3 was the only primary culture expressing IL-6. In response to TGF-β3 stimulation, the levels of IL-6 were notably upregulated in most of the fibroblasts, especially in CAFs.

### Western blot analysis of fibroblast signaling

In parallel, we also analyzed the signaling profile of fibroblasts cultivated in control medium and in medium supplemented with either TGF-β1 or TGF-β3. Representative photographs from the WB analysis are shown in Fig. 1b.

The activation of the canonical TGF-β signaling pathway, as represented by the expression of phospho/total SMAD2/3 in this study, was particularly evident in normal dermal and quiescent scar fibroblasts. Importantly, TGF-β3 treatment attenuated this signaling in keloid and active/hypertrophic scar fibroblasts, while the activation of this signaling pathway remained relatively stable following TGF-β1 treatment. On the other hand, heterogeneous activation of the canonical signaling was observed in CAFs under both control and TGF-β1/3-treated conditions.

Similar expression patterns were observed for SMAD2/3 and β-catenin, a component of the canonical WNT signaling pathway required for TGF-β driven fibrosis, with low levels of expression detected in PANF P10, BCCF B3, and KSF/ASF.

Activation of pAKT was observed in all studied fibroblasts, with the exception of PANF P10. On the other hand, strong pERK signaling was only observed in dermal fibroblasts and BCMFs.

CAFs displayed a diverse response to TGF-β1 and TGF-β3 treatments with regards to AKT and ERK signaling activation. In contrast, only AKT was activated in PANF P10 in response to TGF-β1, with no stimulation observed upon treatment with TGF-β3, making it a particularly resistant primary culture.

The levels of pRAF, RAF, HRAS, and KRAS were also differentially regulated in response to TGF-β1 or TGF-β3 stimulation across different fibroblast types. While TGF-β1 treatment was able to increase levels of pRAF/RAF, TGF-β3 stimulation led to activation of HRAS/KRAS.

Expression of ROCK1 and MCLK was observed in all studied cells under control conditions, except for PANF P10. Following treatment with TGF-β1 or TGF-β3, we observed high heterogeneity in the levels of these proteins, with a prevailing downregulating effect of TGF-β3.

### Immunofluorescence of HDFs, BCCFs, and SCCFs

**Fig. 2:**
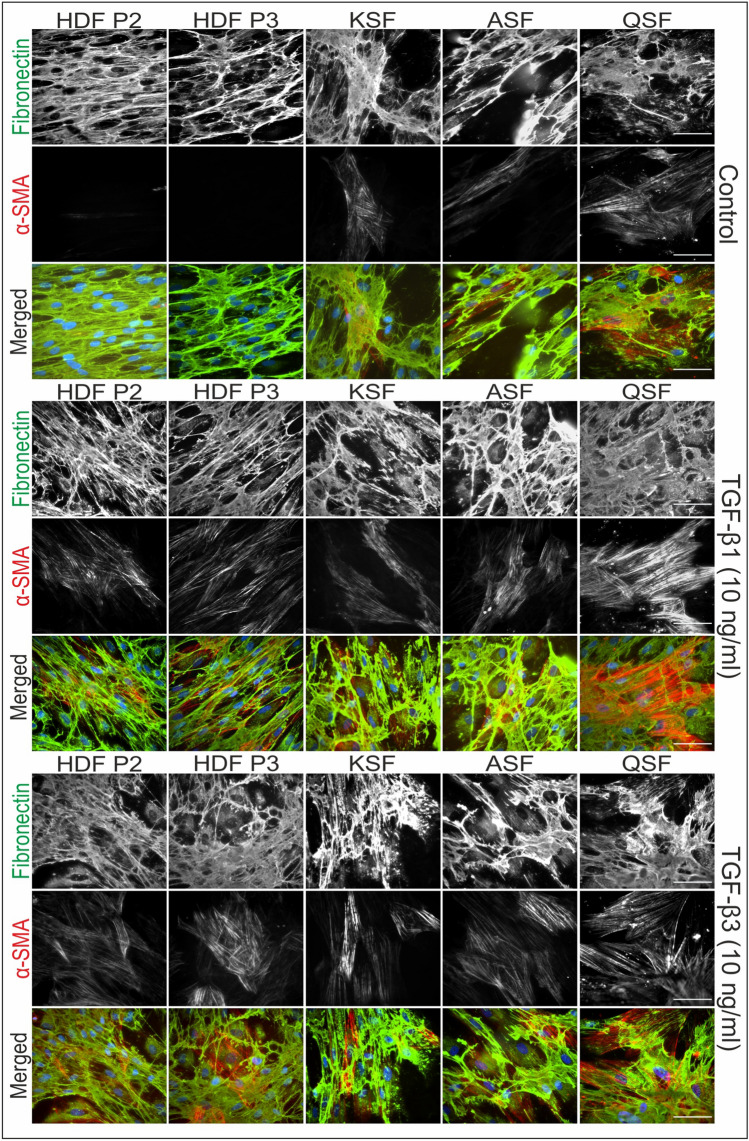
Immunofluorescence analysis of studied fibroblasts derived from skin and scars. Presence of fibronectin and α-smooth muscle actin (α-SMA) in cultures of human dermal fibroblasts (HDFs), keloid scar fibroblasts (KSFs), active/hypertrophic scar fibroblasts (ASFs), and quiescent scar fibroblasts (QSFs). Cell nuclei were counterstained by DAPI. Scale bar 100 µm

**Fig. 3:**
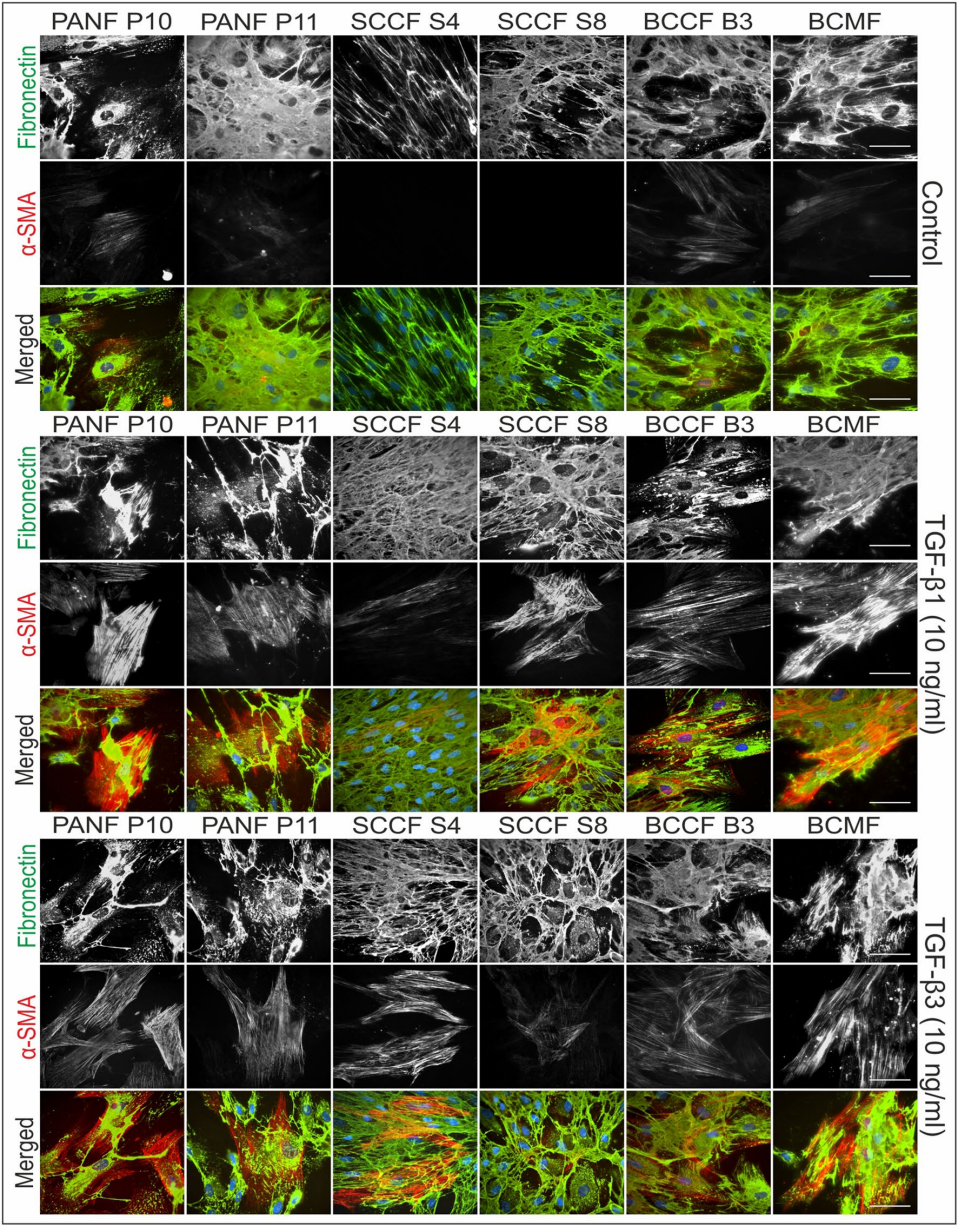
Immunofluorescence analysis of studied cancer-associated fibroblasts. Presence of fibronectin and α-smooth muscle actin (α-SMA) in cultures of pancreatic ductal adenocarcinoma fibroblasts (PANFs), squamous cell carcinoma fibroblasts (SCCFs), basal cell carcinoma fibroblasts (BCCFs), and metastasis of breast adenocarcinoma fibroblasts (BCMFs). Cell nuclei were counterstained by DAPI. Scale bar 100 µm

**Fig. 4:**
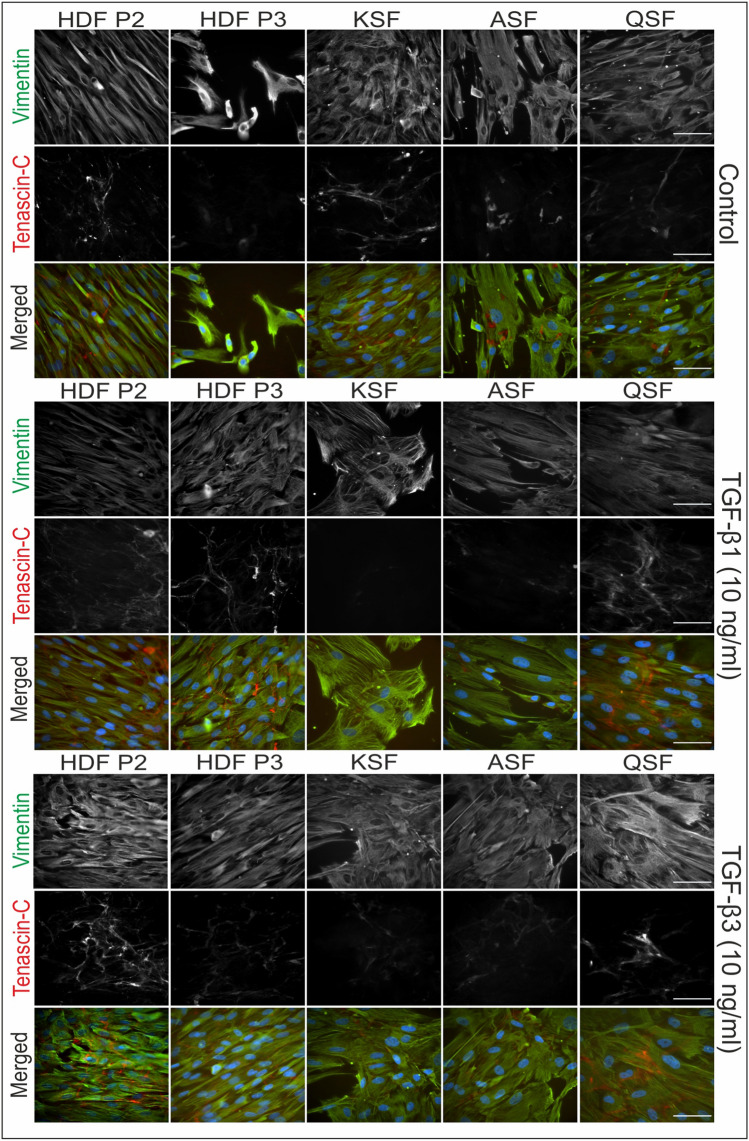
Immunofluorescence analysis of studied fibroblasts derived from skin and scars. Presence of vimentin and tenascin C in cultures of human dermal fibroblasts (HDFs), keloid scar fibroblasts (KSFs), active/hypertrophic scar fibroblasts (ASFs), and quiescent scar fibroblasts (QSFs). Cell nuclei were counterstained by DAPI. Scale bar 100 µm

**Fig. 5:**
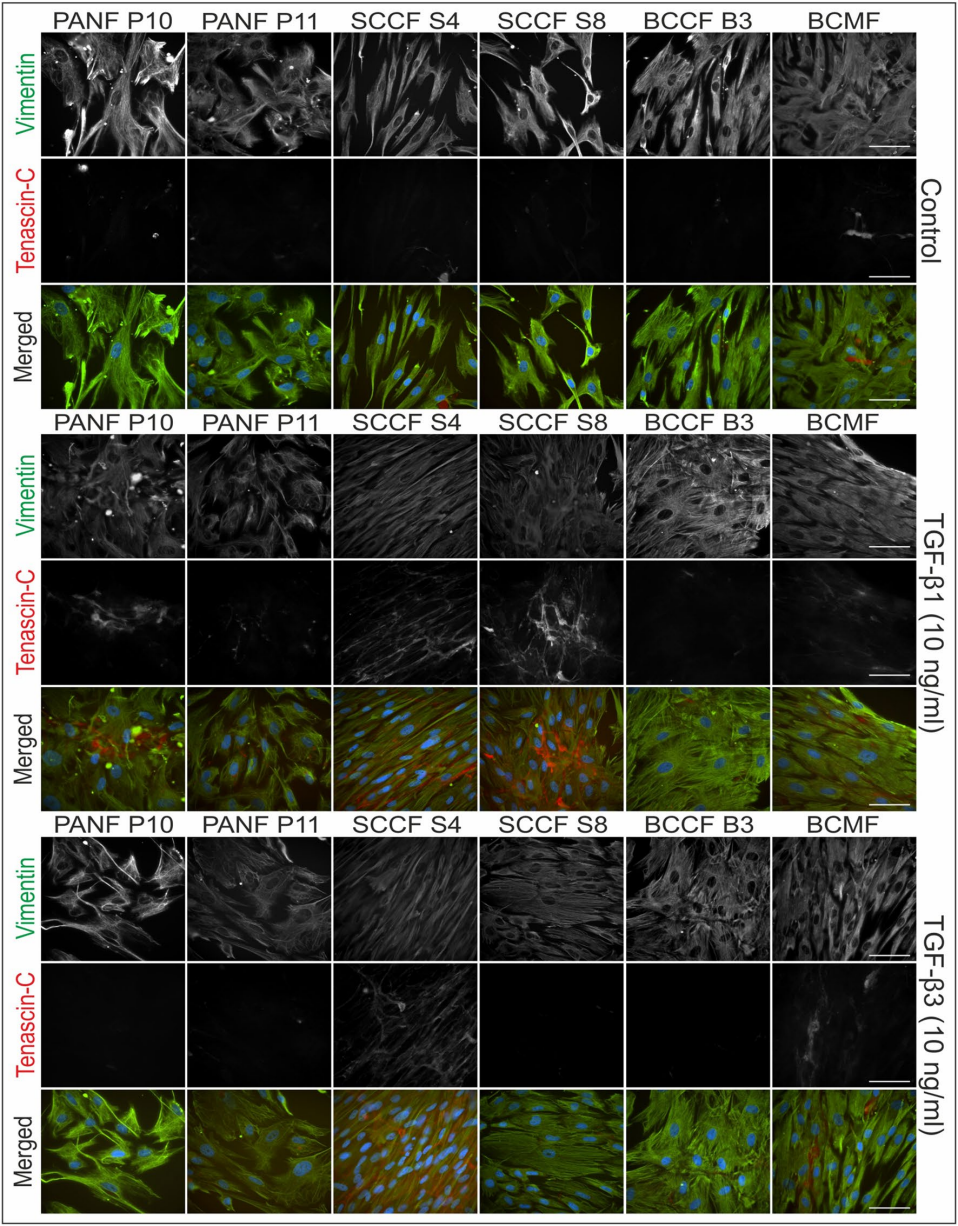
Immunofluorescence analysis of studied cancer-associated fibroblasts. Presence of vimentin and tenascin C in cultures of pancreatic ductal adenocarcinoma fibroblasts (PANFs), squamous cell carcinoma fibroblasts (SCCFs), basal cell carcinoma fibroblasts (BCCFs), and metastasis of breast adenocarcinoma fibroblasts (BCMFs). Cell nuclei were counterstained by DAPI. Scale bar 100 µm

The present study examined the presence of myofibroblasts and fibronectin-rich ECM in various types of fibroblasts. Representative photographs from the IF analysis are shown in Figs. 2 and 3 (fibronectin and α-SMA) and Figs. 4 and 5 (vimentin and tenascin C). The results showed that the expression levels of α-SMA, fibronectin, and tenascin C varied among different fibroblast cell lines and were increased by the presence of TGF-β1 or TGF-β3 in the culture medium (deposition of ECM and presence of myofibroblasts are summarized in Table 3). The presence of vimentin was strong and rather stable in all cultures/conditions.

**Table 3:** Semiquantitative analysis of the presence of myofibroblasts (α-SMA+) and deposited extracellular matrix (fibronectin/tenascin)

	HDF P2	HDF P3	PANF P10	PANF P11	SCCF S4	SCCF S8	BCCF B3	MEBF	KSF	ASF	QSF
α-SMA											
Control	−	−	++	++	−	−	+++	+	+	+	++
TGF-β1	++++	++++	++++	+++	++++	+++	++++	+++	++++	+++	++++
TGF-β3	++++	+++	++++	+++	+++	++	+++	+++	++	++	+++
Fibronectin											
Control	++	+++	+	+	++	++	+	+	+++	++	++
TGF-β1	+++	++++	++	+	++++	+++	+	+	+++	++	++
TGF-β3	+++	+++	++	++	++++	+++	+	+	+++	++	++
Tenascin C											
Control	+	+	−	+	+	+	−	−	+	−	+
TGF-β1	++++	++++	++	+	+++	+++	−	++	++	+	++
TGF-β3	++++	++++	+	−	++++	+++	−	+	++	−	++

The vast majority of BCCF B3 cell expressed α-SMA-positive myofibroblast-like phenotype. Myofibroblast-like cells were also present, to a lesser extent, in cultures of PANFs, BCMFs, and scar-derived fibroblast. Interestingly, myofibroblasts in KSF cultures were observed to be concentrated in nests rather than being dispersed. Following treatment with TGF-β1 and TGF-β3, a strong upregulation of α-SMA expression was observed, indicating the induction of fibroblast-to-myofibroblast switch. However, in direct comparison the effect of TGF-β1 was even stronger.

In contrast to the differential effects of TGF-β1 and TGF-β3 on α-SMA induction, treatment with TGF-β1 and TGF-β3 had almost identical effects on fibronectin (ECM) deposition. Specifically, both TGF-β1 and TGF-β3 led to increased fibronectin deposition in HDFs, PANFs, and SCCFs, while other cell cultures remained largely unaffected. The expression of the other examined ECM marker, tenascin C, was remarkably weaker in all cultures. Only HDFs and SCCFs were markedly responsive to TGF treatments whereas the other CAFs and scar-derived cells responded to a lesser extent. Of note, BCCFs remained tenascin C-negative in all conditions.

## Discussion

Here, we report our findings supporting the critical roles of TGF-β signaling in the regulation of protein expression and cell differentiation in several types of fibroblasts, including normal dermal, cancer-associated, and scar-derived fibroblasts. Our results revealed that myofibroblast presence and ECM deposition were dependent on the origin of the fibroblasts and the type of treatment they received (TGF-β1 vs. TGF-β3). Myofibroblasts are cells that play a critical role in fibrotic diseases and cancer (Pakshir et al. 2020). Therefore, we examined the crucial aspects of signaling by comparing expression profiles of proteins within the canonical and non-canonical pathways (Turati et al. 2023). Upregulation of pSMAD2/3, a key component of the canonical TGF-β signaling pathway (Turati et al. 2023), was particularly evident in normal dermal and quiescent scar fibroblasts. Regarding potential clinical relevance, rather decreased signaling was noted following cell treatment with TGF-β3 in keloid and active/hypertrophic scar fibroblasts. Similar expression patterns were observed for SMAD2/3 and β-catenin, a component of the canonical WNT signaling pathway required for TGF-β driven fibrosis (Akhmetshina et al. 2012; Xu et al. 2017). It has been shown that simultaneous neutralizing of both TGF-β1 and TG-Fβ2 or supplementing with TGF-β3 in cutaneous rat wounds improved neodermis architecture and decreased scarring (Shah et al. 1995). However, blocking TGF-β1 alone reduced the monocyte and macrophage profile, fibronectin, collagen III, and collagen I deposition, but only marginally reduced scarring; blocking TGF-β2 alone did not show any significant improvement. Although targeting the expression of TGF-β1 or TGF-β2 is unlikely to result in scarless healing (Penn et al. 2012), the level of TGF-β1 correlated well with the severity of scarring (Deng et al. 2021).

Furthermore, the canonical TGF-β signaling has been also implicated in fibroblast proliferation, as demonstrated by studies showing increased SMAD2/3 phosphorylation in response to TGF-β1 stimulation (Meran et al. 2008). In fact, SMAD3 has been shown to be important for fibroblast proliferation, as fibrotic lesions in Smad3-deficient mice have fewer myofibroblasts (Lakos et al. 2004). TGF-β signaling also activates PI3K-AKT-p21-activated kinase-2 signaling in AKR-2B murine fibroblasts, leading to increased fibroblast proliferation (Wilkes et al. 2005). Moreover, studies have shown that TGF-β non-canonical signaling pathways are involved in the apoptosis-resistant properties of myofibroblasts associated with fibrosis, which cannot revert to a quiescent state (Kalluri 2016; Thannickal and Horowitz 2006; Yoshida et al. 2002). In human lung fibroblasts, TGF-β1 activates PI3K/AKT via the p38 MAPK pathway, which protects them from apoptosis induced by the Fas-caspase cascade (Kulasekaran et al. 2009). These findings suggest that both canonical and non-canonical TGF-β signaling pathways contribute to fibroblast proliferation and apoptosis-resistant properties in fibrotic diseases.

In parallel, the non-canonical signaling pathways can drive fibroblast-to-myofibroblast transition independently of SMADs. Upon TGF-β stimulation of human skin fibroblasts, phosphorylation of MAP-kinase ERK1/2 is consistently elevated, which promotes differentiation of fibroblasts via the upregulation of transcription factor FRA2, a downstream mediator of TGF-β with a pro-fibrotic effect (Carthy et al. 2015; Reich et al. 2010; Eferl et al. 2008). Inhibiting ERK1/2 activation with a MEK1/2 inhibitor can attenuate TGF-β-mediated activation of myofibroblasts without affecting pSMAD2 or pSMAD3 (Carthy et al. 2015). Disrupting Rho signaling also attenuates myofibroblast transformation and collagen synthesis in human lung fibroblasts (Ni et al. 2013). In a mouse in vitro model using Swiss3T3 fibroblasts, activated TβRI stimulated Rho GTPases via Rho-Rho Kinase 1-LIM-kinase 2 phosphorylation that inactivated the actin-depolymerizing factor cofilin. SMAD2/3 mutation did not affect actin remodeling or Rho activation, indicating that non-canonical Rho signaling components, but not canonical signaling, are responsible for actin formation and reorganization.

We detected activation of canonical signaling (pSMAD2/3) across all studied fibroblast, albeit to various extents. Treatment with TGF-β1 and TGF-β3 resulted in activation of canonical and several non-canonical pathways, including AKT, ERK, RAF, and ROCK preferably in normal dermal fibroblast. In contrast, CAFs displayed rather characteristic heterogenic response to TGF-β1/3 treatments. TGF-β1 demonstrated a more potent activation of signaling pathways compared to TGF-β3. Intriguingly, TGF-β3 exhibited rather an opposite effect by reducing both canonical and non-canonical signaling in KS and AS derived fibroblasts, suggesting potential clinical implications.

Identification of CAFs has typically relied on the expression of various “CAF markers” such as fibroblast activation protein alpha (FAP) and α-SMA, which differentiate them from the larger pool of fibroblasts present in the body. However, the expression of commonly used fibroblast markers is highly heterogeneous and varies significantly between different CAF subpopulations (Nurmik et al. 2020). We observed extreme heterogeneity of studied markers across different fibroblast populations, even within those isolated from the same tissue. None of the markers showed clear specificity for normal dermal, scar-derived, or cancer-associated fibroblasts, challenging the precise definition of the heterogeneous CAF populations.

Interestingly, we found high α-SMA expression in quiescent scar fibroblasts, whereas keloid and active/hypertrophic scar fibroblasts did not exhibit this characteristic. This finding suggests the absence of myofibroblasts in these particular populations of scar fibroblasts. The single-cell sequencing analysis of various normal and pathological scar tissues identified four distinct fibroblast subtypes: mesenchymal, pro-inflammatory, secretory-papillary, and secretory-reticular. Importantly, these subtypes exhibited varying levels of myofibroblast presence, ranging from 7.6% to 53.8% (Deng et al. 2021). Specifically, the presence of myofibroblasts was increased in keloids compared to normal scars, with approximately 26% versus 13% prevalence, respectively. However, as a result of limitations of the isolation technique (Dvorankova et al. 2012) used in our study we were unable to clonally expand specific fibroblast subpopulations. Instead, we isolated cells with varying prevalence of specific subpopulations. Notably, we observed similar expression patterns of F-actin and α-SMA. The shift in G- to F-actin ratio liberates myocardin-related transcription factor A from its inhibitory complex with G-actin in the cytoplasm and allows its translocation to the nucleus driving the transcription of profibrotic gene products, such as CCN2 and α-SMA (Speight et al. 2016; Varney et al. 2016).

Although FAP has been widely considered a CAF-specific marker (Shi et al. 2012; Han et al. 2020), we found that it was strongly expressed in normal dermal and scar-derived fibroblasts. The only observable trend in marker expression was strong expression of PDGFR-α and -β in normal fibroblasts isolated from dermis, while CAF and scar-derived fibroblasts expressed lesser amounts of these receptors. To elucidate which CAF subpopulations exert a pro- versus antitumor effect, it is crucial to build upon precise knowledge of CAF markers and their functions (LeBleu and Kalluri 2018). Understanding the heterogeneity of CAFs and their roles in cancer progression will be critical for the development of targeted therapies that selectively modulate specific CAF subpopulations.

Single-cell sequencing of pancreatic ductal adenocarcinoma and breast carcinoma samples revealed the presence of two main phenotypically distinct populations of CAFs, namely myofibroblastic-like CAFs (myCAFs) and inflammatory-like CAFs (iCAFs) (Elyada et al. 2019; Kieffer et al. 2020). While myCAFs were mainly involved in extracellular matrix remodeling, iCAFs produced pro-inflammatory cytokines, including IL-6, an important player in cancer progression and wound healing (Kumari et al. 2016; Gal et al. 2022). Intriguingly, we found that BCCF B3, the only fibroblast population producing high amounts of IL-6 in control conditions, also expressed high amounts of α-SMA, with the highest presence of myofibroblasts observed by IF across all studied fibroblasts in control conditions. Treatment with TGF-β3 was able to induce IL-6 production in various fibroblast populations, whereas TGF-β1 treatment led to attenuation of IL-6 production in BCCF B3 cells. These findings suggest a potential role of TGF-β3 in driving the iCAF phenotype transition. Notably, the secreted form of IL-6 is not detectable through standard WB analysis which represents a certain limitation of this study. We postulate that the detected expression of IL-6 is associated with its accumulation in the cell cytoplasm as a result of overproduction. In contrast, tenascin C, another soluble factor, binds to fibronectin (Midwood et al. 2016) and can be detected in the deposited ECM by WB analysis. Our study supports this notion as we observed a correlation between tenascin C and fibronectin expression across all investigated conditions.

## Conclusion

Our study highlights the heterogeneity of fibroblast populations and their varying functions in the tumor and wound microenvironments. We clearly demonstrated that TGF-β signaling engages not only canonical pathways but also multiple non-canonical pathways, thereby underscoring the heterogeneity of fibroblasts, which may be influenced by specific clinical conditions. Furthermore, our findings suggest that the responsiveness and behavior of fibroblasts in different clinical contexts may vary, potentially impacting therapeutic outcomes. Understanding the specific roles of diverse fibroblast populations and the TGF-β signaling pathways they engage in various clinical conditions is crucial for developing targeted interventions and optimizing treatment strategies.

## Data Availability

The datasets generated and analyzed during the current study are available from the corresponding authors on reasonable request.
